# 48-year-old Man with Fevers, Chest Pain, and a History of Substance Abuse

**DOI:** 10.5811/cpcem.2018.8.39252

**Published:** 2018-10-08

**Authors:** Adam Richardson, Semhar Z. Tewelde, Zachary D.W. Dezman

**Affiliations:** University of Maryland School of Medicine, Department of Emergency Medicine, Baltimore, Maryland

## Abstract

A 48-year-old male with a history of intravenous (IV) drug use presented to the emergency department (ED) for an area of mild pain and erythema on his chest. He was then triaged to the urgent care, or fast track, area of the ED. He was well appearing with normal lab findings and vital signs, but his workup revealed mediastinitis with osteomyelitis of the manubrium and clavicles, a surgical emergency. His treatment course included IV antibiotics and operative intervention with thoracic surgery. The patient *looked too good to be sick*, yet he had a life-threatening infection.

## INTRODUCTION

Intravenous (IV) drug abuse is a risk factor for several insidious and life-threatening infections seen in the emergency department (ED). This case describes our experience treating one such infection: mediastinitis. We also discuss the physical exam findings associated with this disease.

## CASE REPORT

**Image 1:**
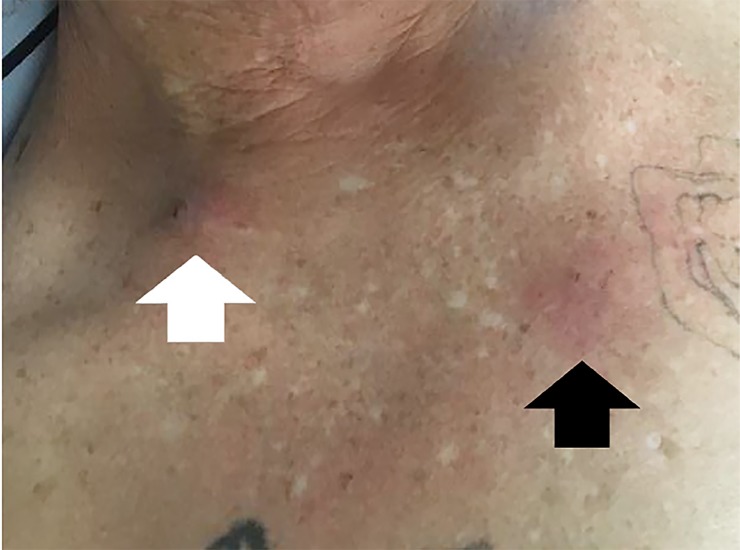
Skin findings as seen while the patient is sitting upright (base of neck at top of image). Note the puncture mark within the supraclavicular space (white arrow) and the erythema across the sternum (black arrow).

**Image 2:**
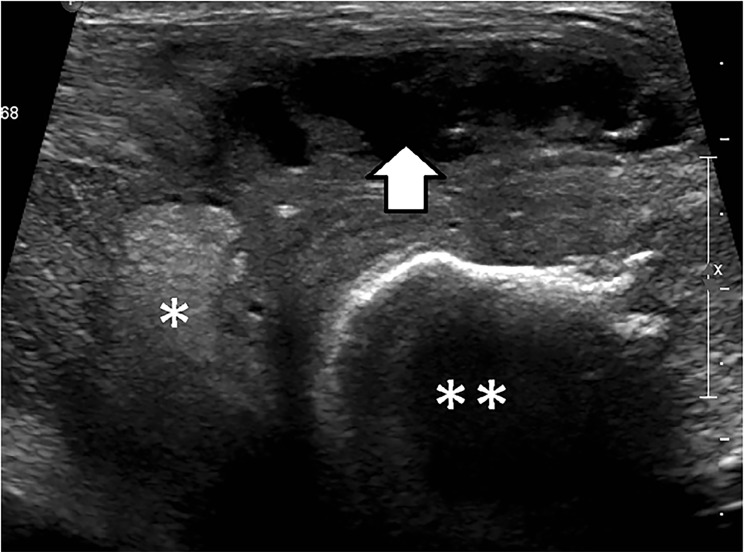
Ultrasound image of the area of pain and erythema between the sternum (*) and the right clavicle (**), demonstrating a subcutaneous fluid collection (arrow).

**Image 3:**
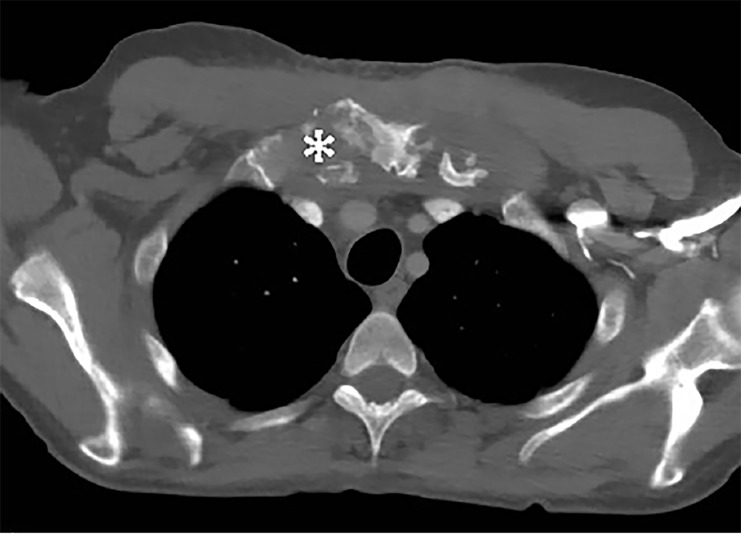
Transverse computed tomography image of the chest, taken at the level of the manubrium, showing widespread destruction of the bony tissue (*).

A 48-year-old male with a history of polysubstance abuse came to the urgent care area of our ED with a complaint of constant, aching pain over his sternum and right clavicle. The pain had gradually worsened and was accompanied by intermittent subjective fevers over the prior week. That day the area developed erythema, swelling, and fluctuance (Image 1). The patient was afebrile and had a pulse of 89 beats per minute, a blood pressure of 116/70 milligrams of mercury, a respiratory rate of 16 breaths per minute, and 100% pulse oximetry on room air. He was well appearing on exam, requesting food, and he frequently left to smoke cigarettes outside. The patient had a normal white blood cell count and venous lactate. An ultrasound of the area of pain and swelling (Image 2) and a computed tomography (CT) of the patient’s chest (Image 3) were completed.

The preliminary ultrasound report revealed an abscess adjacent to the patient’s right sternoclavicular joint (Image 2). A CT of the chest revealed bony destruction of the manubrium and clavicles with abscess extending into the anterior mediastinum (Image 3). The patient was admitted for IV antibiotics and underwent a bilateral sternoclavicular debridement and abscess drainage with thoracic surgery.

## DISCUSSION

Septic arthritis and osteomyelitis of the manubrium is most commonly associated with IV drug use (21%), often a result of patients using non-sterile technique to access their internal jugular or subclavian veins to inject illicit substances using a supraclavicular approach (“pocket shot”).^1^,^2^ Other risk factors include infections at a distant site (15%), diabetes (13%), trauma (12%), and infected central venous access (9%).^1^,^2^ Local cellulitis progresses to an abscess and septic arthritis, leading to osteomyelitis and mediastinitis, as seen in this patient. Mediastinitis is rare in the current era of antibiotics, with complications from cardiac surgery, esophagogastroduodenoscopy, or suppurative infections of the head and neck being the most common causes.^3^ Patients present after several weeks of chest pain, fevers, and dyspnea.^3^ Inflammatory markers like C-reactive protein, erythrocyte sedimentation rate, and procalcitonin will be elevated in these patients and imaging should include CT of the chest with contrast.^4^,^5^ Blood cultures should be drawn prior to the administration of broad-spectrum antibiotics, with the causative organism being Gram-positive (*Staphylococcus aureus*) more often than Gram-negative (*Pseudomonas aeruginosa*).^2^ Thoracic surgery should be consulted on these patients as most require surgical abscess drainage.^1^,^5^

**CPC-EM Capsule**

What do we already know about this clinical entity?

Intravenous drug use is a known risk factor for devastating and sometimes subtle infections like medistinitus.

What makes this presentation of disease reportable?

This case links the easily-overlooked physical features of a well-appearing patient with their ultrasound and computed tomography results showing an extensive case of mediastinitis.

What is the major learning point?

Patients who are functionally immune-suppressed, like those who abuse substances intravenously, are at high risk for cryptic and devastating infections.

How might this improve emergency medicine practice?

Readers will be able to better recognize and screen patients at high-risk for these infections.

## CONCLUSION

This case report is an excellent example of someone who *looked too good to be sick*, yet his workup revealed an impressive and extensive infectious process that required emergency surgery. His presentation was innocuous as he did not have a toxic appearance; and his vital signs, white blood cell count, and serum lactate were within normal limits. The only abnormalities in his presentation were the area of mild erythema and fluctuance, although the area was only mildly tender and he appeared comfortable. Initially only basic labs and a limited ultrasound study were ordered because the initial presentation did not seem to warrant CT, blood cultures or other more extensive measure. But once the ultrasound revealed an abscess adjacent to the sternoclavicular joint, the complete workup was ordered.

As primary care offices, urgent care centers, and EDs across the nation become increasingly overwhelmed, it is often difficult to justify an extensive workup for a person who looks well and has no demonstrable signs of a worrisome infection. A clinician who is less experienced with invasive soft tissue infections could have reasonably diagnosed this man with cellulitis and a superficial abscess and elected to treat him with outpatient antibiotics, which would not have been effective. By convincing the patient to be forthcoming regarding injection drug use, we were able to add serious infectious process to the differential diagnosis even in the absence of classic symptoms, and that was the key to making the correct diagnosis in a timely manner.

Documented patient informed consent and/or Institutional Review Board approval has been obtained and filed for publication of this case report.
